# Cross-sectional associations between cardiorespiratory fitness and NMR-derived metabolic biomarkers in children – the PANIC study

**DOI:** 10.3389/fendo.2022.954418

**Published:** 2022-09-23

**Authors:** Eero A. Haapala, Marja H. Leppänen, Maarit Lehti, Niina Lintu, Tuomo Tompuri, Anna Viitasalo, Ursula Schwab, Timo A. Lakka

**Affiliations:** ^1^ Faculty of Sport and Health Sciences, University of Jyväskylä, Jyväskylä, Finland; ^2^ Institute of Biomedicine, School of Medicine, University of Eastern Finland, Kuopio, Finland; ^3^ Folkhälsan Research Center, Helsinki, Finland; ^4^ Department of Clinical Physiology and Nuclear Medicine, Kuopio University Hospital, Kuopio, Finland; ^5^ Institute of Public Health and Clinical Nutrition, School of Medicine, University of Eastern Finland, Kuopio, Finland; ^6^ Department of Medicine, Endocrinology and Clinical Nutrition, Kuopio University Hospital, Kuopio, Finland; ^7^ Foundation for Research in Health Exercise and Nutrition, Kuopio Research Institute of Exercise Medicine, Kuopio, Finland

**Keywords:** fitness, aerobic fitness, metabolism, metabolomics, pediatrics, obesity, lipoproteins

## Abstract

**Objective:**

Cardiorespiratory fitness has been inversely associated with cardiovascular risk across the lifespan. Some studies in adults suggest that higher cardiorespiratory fitness is associated with cardioprotective metabolite profile, but the evidence in children is lacking. Therefore, we investigated the cross-sectional association of cardiorespiratory fitness with serum nuclear magnetic resonance derived metabolic biomarkers in children.

**Methods:**

A population sample of 450 children aged 6–8 years was examined. Cardiorespiratory fitness was assessed by a maximal exercise test on a cycle ergometer and quantified as maximal power output normalised for lean body mass assessed by dual-energy X-ray absorbtiometry. Serum metabolites were assessed using a high throughput nuclear magnetic resonance platform. The data were analysed using linear regression analyses adjusted for age and sex and subsequently for body fat percentage (BF%) assessed by DXA.

**Results:**

Cardiorespiratory fitness was directly associated with high density lipoprotein (HDL) cholesterol (β=0.138, 95% CI=0.042 to 0.135, p=0.005), average HDL particle diameter (β=0.102, 95% CI=0.004 to 0.199, p=0.041), and the concentrations of extra-large HDL particles (β=0.103, 95% CI=0.006 to 0.201, p=0.038), large HDL particles (β=0.122, 95% CI=0.025 to 0.220, p=0.014), and medium HDL particles (β=0.143, 95% CI=0.047 to 0.239, p=0.004) after adjustment for age and sex. Higher cardiorespiratory fitness was also associated with higher concentrations of ApoA1 (β=0.145, 95% CI=0.047 to 0.242, p=0.003), glutamine (β=0.161, 95% CI=0.064 to 0.257, p=0.001), and phenylalanine (β=0.187, 95% CI=0.091 to 0.283, p<0.001). However, only the direct associations of cardiorespiratory fitness with the concentrations of HDL cholesterol (β=0.114, 95% CI=0.018 to 0.210, p=0.021), medium HDL particles (β=0.126, 95% CI=0.030 to 0.223, p=0.010), ApoA1 (β=0.126, 95% CI=0.030 to 0.223, p=0.011), glutamine (β=0.147, 95% CI=0.050 to 0.224, p=0.003), and phenylalanine (β=0.217, 95% CI=0.122 to 0.311, p<0.001) remained statistically significant after further adjustment for BF%.

**Conclusions:**

Higher cardiorespiratory fitness was associated with a cardioprotective biomarker profile in children. Most associations were independent of BF% suggesting that the differences in serum metabolites between children are driven by cardiorespiratory fitness and not adiposity.

## Introduction

Low cardiorespiratory fitness has been associated with increased risk for cardiovascular diseases and cardiovascular events in adults (1, 2). Nevertheless, pathophysiological processes for atherosclerotic cardiovascular diseases start already in childhood. Accordingly, the early signs of atherosclerotic cardiovascular diseases such as increased arterial stiffness (3, 4) and increased carotid intima media thickness and distensibility (4, 5), have been detected already in children and adolescents. In addition, some evidence suggests that increasing cardiorespiratory fitness since youth could stagnate the development of early signs of atherosclerotic cardiovascular diseases over the life course (6). Nevertheless, cardiorespiratory fitness has been found to have weak if any associations with traditional cardiovascular disease risk factors, such as insulin resistance, dyslipidaemia, and blood pressure in children (7–10), leaving a knowledge gap on how cardiorespiratory fitness could contribute to cardiovascular health in a general population of children. Thus, while cardiorespiratory fitness may protect against atherosclerotic cardiovascular diseases, the mechanisms are not well understood, especially in children and more studies are warranted. Serum metabolomics provides a novel approach to deepen our understanding of the mechanisms underlying the health benefits of cardiorespiratory fitness.

Impaired lipid metabolism, such as increased low-density lipoprotein (LDL) cholesterol concentration and apolipoprotein B (ApoB), is well-known risk factor for atherosclerotic cardiovascular diseases (11). Metabolomics can provide further characterisation of lipids improving the cardiovascular risk estimation (12). For example, increased very-low-density lipoprotein (VLDL) concentration and reduced high density lipoprotein (HDL) concentration and size have been associated with an increased risk of atherosclerotic cardiovascular diseases (13–16). Furthermore, traditional lipid biomarkers, such as LDL cholesterol, HDL cholesterol, and triglycerides were not associated with carotid atherosclerosis or carotid-femoral pulse wave velocity, whereas a total HDL particle concentration and average HDL size were inversely associated with them in obese youth aged 18 years (17). The studies utilising metabolomics have also found that increased concentrations of branched-chain amino acids are associated with increased risk of atherosclerotic cardiovascular diseases (15, 18) and increased carotid artery intima media thickness, and exercise induced myocardial ischaemia (19) in adults. However, it should be noted that there are only few studies on the associations between metabolites and the measures of cardiovascular health among youth. However, one study found that none of the investigated metabolites were associated with carotid intima-media thickness or pulse wave velocity after adjustment for body mass index and blood pressure in children (20).

Cardiorespiratory fitness has been inversely associated with lipid metabolites and branched-chain amino acids in adults (21), but there are paucity of data in children. Nevertheless, one small study in adolescents found that cardiorespiratory fitness was inversely associated with amino acids valerate, glutamate, and tyrosine (22). However, cardiorespiratory fitness was assessed by maximal oxygen uptake (V̇O_2max_) normalised for whole body mass, a measure of cardiorespiratory fitness strongly confounded by adiposity (23). Similarly, another study in young adults showed that higher cardiorespiratory fitness quantified, as estimated V̇O_2max_ normalised for whole body mass, was inversely associated with the concentrations of extra-large to small size VLDL, large to small size LDL, ApoB, and ApoB to apolipoprotein A1 (ApoA1) ratio and directly associated with the concentrations of extra-large to medium size HDL and ApoA1 (24). Furthermore, cardiorespiratory fitness has been inversely associated with branched-chain amino acids and phenylalanine, tyrosine and positively associated with glutamine (24). However, when body fat percentage (BF%) was controlled for, cardiorespiratory fitness was associated only with the concentrations of large HDL, ApoA1, and medium size VLDL (24) suggesting that adiposity is a strong confounder in these associations.

There is limited evidence on the associations of cardiorespiratory fitness with serum nuclear magnetic resonance (NMR) derived metabolic biomarkers in children, although pathophysiological process leading to atherosclerotic cardiovascular diseases often begin in childhood. Therefore, we first investigated the associations of cardiorespiratory fitness with serum NMR-derived biomarkers related to lipoproteins, triglycerides, apolipoproteins, and amino acids in a general population of children. Second, we investigated whether BF% modifies these associations. Finally, because HDL characteristics potentially have specific effects on cardiovascular health and the determinants of cardiorespiratory fitness (25–27), we also investigated the associations of cardiorespiratory fitness with various HDL characteristics.

## Methods

### Participants

The present cross-sectional data are from the baseline assessments of the Physical Activity and Nutrition in Children (PANIC) Study, which is an 8-year physical activity and dietary intervention study and a long-term follow-up study in a population sample of children from the city of Kuopio, Finland (28). The Research Ethics Committee of the Hospital District of Northern Savo approved the study protocol in 2006 (Statement 69/2006). The parents or caregivers of the children gave their written informed consent, and the children provided their assent to participation. The PANIC study has been carried out in accordance with the principles of the Declaration of Helsinki as revised in 2008.

Altogether 736 children 6–8 years of age from primary schools of Kuopio were invited to participate in the baseline examination in 2007–2009. A total of 512 children, who represented 70% of those invited, participated in the baseline examinations. Six children were excluded from the study at baseline because of physical disabilities that could hamper participation in the intervention or no time or motivation to attend in the study. The participants did not differ in sex distribution, age, or body mass index standard deviation score (BMI-SDS) from all children who started the first grade in 2007–2009 based on data from the standard school health examinations performed for all Finnish children before the first grade (data not shown). Complete data on variables used in the analyses on the associations of cardiorespiratory fitness with serum metabolites were available for 450 children (217 girls, 233 boys).

### Assessment of body size and composition

Whole body mass was measured twice with the children having fasted for 12 hours, emptied the bladder, and standing in light underwear by a calibrated InBody^®^ 720 bioelectrical impedance device (Biospace, Seoul, South Korea) to an accuracy of 0.1 kg. The mean of these two values was used in the analyses. Stature was measured three times with the children standing in the Frankfurt plane without shoes using a wall-mounted stadiometer to an accuracy of 0.1 cm. The mean of the nearest two values was used in the analyses. BMI was calculated by dividing body mass (kg) by body height (m) squared. BMI-SDS was calculated based on Finnish reference data (29). The prevalence of overweight and obesity was defined using the cut-off values provided by Cole et al. (30). Total fat mass, BF%, and lean mass (LM) were measured by the Lunar^®^ dual-energy X-ray absorptiometry device (GE Medical Systems, Madison, WI, USA) using standardised protocols (31).

### Assessment of cardiorespiratory fitness

We assessed cardiorespiratory fitness by a maximal exercise test using an electromagnetically braked Ergoselect 200 K^®^ cycle ergometer coupled with a paediatric saddle module (Ergoline, Bitz, Germany) (32). The exercise test protocol included a 2.5-minute anticipatory period with the child sitting on the ergometer; a 3-minute warm-up period with a workload of 5 watts; a 1-minute steady-state period with a workload of 20 watts; an exercise period with an increase in the workload of 1 watt per 6 seconds until exhaustion, and a 4-minute recovery period with a workload of 5 watts.

The children were asked to keep the cadence stable and within 70–80 revolutions per minute. Exhaustion was defined as the inability to maintain the cadence above 65 revolutions per minute regardless of vigorous verbal exhortation. The exercise test was considered maximal by an experienced physician (TT) supervising the test, if objective and subjective criteria (heart rate >85% of predicted, sweating, flushing, inability to continue exercise test regardless of strong verbal encouragement) indicated maximal effort and maximal cardiovascular capacity. Heart rate was measured continuously during the last five minutes of the supine rest prior to commencing the exercise test protocol right through to the 5-minute supine post-exercise rest period using a 12-lead electrocardiogram registered by the Cardiosoft^®^ V6.5 Diagnostic System (GE Healthcare Medical Systems, Freiburg, Germany) and the highest heart rate during the test was defined as peak heart rate. Maximal power output (W_max_) measured at the end of the exercise test divided by LM^-1^ were used as a measure of cardiorespiratory fitness. We used W_max_ as a measure of cardiorespiratory fitness because we did not perform respiratory gas analyses at baseline and it has been found to be a good surrogate measure of cardiorespiratory fitness in children (33). W_max_ x kg of LM^-1^ was no associated with LM (β=0.072, 95% CI=-0.013 to 0.156) and the sex interaction was not statistically significant (p=0.235) suggesting the validity of the scaling procedure.

### Assessment of metabolic biomarkers

A research nurse took blood samples in the morning, after children had fasted overnight for at least 12 hours. Blood was immediately centrifuged and stored at a temperature of -75°C until biochemical analyses. The Nightingale high-throughput NMR platform was used to quantify serum metabolic biomarkers (34). The Nightingale NMR platform quantifies different metabolic biomarkers in absolute concentration units. Based on the aims of the study and previous literature, we selected metabolites related to lipoprotein, triglyceride, apolipoprotein, and amino acid metabolism and specifically focused on 49 HDL characteristics.

### Assessment of confounding factors

The research physician assessed pubertal status using the 5-stage scale described by Tanner (35, 36). The boys were defined as having entered clinical puberty if their testicular volume assessed by an orchidometer was ≥4 mL (stage ≥2). The girls were defined having entered clinical puberty if their breast development had started (stage ≥2). Maturity was assessed as the difference between the current age from the age at predicted peak height velocity and it was computed using a sex-specific formula described by Moore et al. (37). Physical activity energy expenditure (PAEE) and time accumulated in moderate to vigorous physical activity (MVPA) were assessed by a combined heart rate and movement monitor (38). We included all children who had device-assessed data on physical activity regardless of wearing time requirements used in our previous studies (38, 39). The mean (standard deviation, SD) wear time was 113 (40) hours for the 402 children who met the wear time requirements and 61 (33) hours for the 33 children who did not meet these requirements. PAEE, MVPA, BF%, cardiorespiratory fitness, maturity, or diet quality did not differ between these two groups (p>0.290). Diet quality was assessed by four day dietary records (28), and the overall diet quality was computed using the Finnish Children Healthy Eating Index (FCHEI) (41). Homeostatic Model Assessment of Insulin Resistance (HOMA-IR) as a measure of insulin resistance was computed from the fasting plasma glucose and serum insulin as prescribed earlier (42). Missing data were replaced using the sample mean values.

### Statistical methods

The analyses were performed using the Jamovi statistical software, version 2.2 (Jamovi project 2021). First, we investigated the differences in basic characteristics between girls and boys using the Welch’s -test for normally distributed continuous variables, the Mann-Whitney U -test for continuous variables with skewed distributions, and the χ^2^ test for categorical variables. Second, we studied the associations of cardiorespiratory fitness with metabolites using the linear regression analyses adjusted for age and sex (Model 1) and additionally for BF% (Model 2). We also investigated whether sex or BF% modified the associations of cardiorespiratory fitness with serum metabolites by adding the interaction term of sex or BF% and cardiorespiratory fitness into the models. If the interaction was statistically significant, we analysed the associations of cardiorespiratory fitness with metabolites separately among children below and at or above the sex-specific median of BF% or among girls and boys. The data were corrected for multiple comparisons using the Benjamini-Hochberg false discovery rate (FDR) using the FDR value of 0.2 (FDR_0.2_) and 0.1 (FDR_0.1_). The data were further adjusted for maturity, PAEE, MVPA, FCHEI, or HOMA-IR. The data on the associations between cardiorespiratory fitness and amino acids were further adjusted for protein intake as a percentage of total energy. We did not adjust the data for clinical puberty because few children had signs of clinical puberty.

## Results

### Basic characteristics

Girls were shorter and more likely to have entered clinical puberty and had advanced maturity, higher BF%, and lower LM than boys (**Table 1**). Girls also had lower cardiorespiratory fitness and accumulated less PAEE and MVPA than boys.

**Table 1:** Characteristics of participants.

	All children (n=450)	Girls (n=217)	Boys (n=233)	p
Age (years)	7.6 (0.4)	7.6 (0.4)	7.7 (0.4)	0.362
Stature (cm)	129 (5.5)	128 (5.7)	130 (5.2)	**<0.001**
Body weight (kg)	26.0 (23.6 to 29.3)	25.5 (23.1 to 29.0)	26.6 (23.9 to 29.4)	0.059
Clinical puberty (%)	2.5	4.3	0.8	**0.031**
Maturity (years)	-4.1 (-4.4 to -3.6)	-3.6 (0.3)	-4.4 (0.3)	**<0.001**
Body mass index standard deviation score	-0.18 (1.1)	-0.15 (1.1)	-0.20 (1.1)	0.642
Prevalence of overweight (%)	13.1	15.2	11.2	0.204
Body fat percentage (%)	18.7 (13.3 to 24.2)	20.8 (17.4 to 27.0)	15.0 (11.4 to 21.5)	**<0.001**
Lean body mass (kg)	20.6 (2.4)	19.5 (2.1)	21.6 (2.2)	**<0.001**
Homeostatic Model Assessment of Insulin Resistance				
Absolute peak power output (W)	76.7 (15.3)	69.9 (12.9)	82.9 (14.6)	**<0.001**
Relative peak power output (W x kg of LM^-1^)	3.7 (0.5)	3.6 (0.5)	3.8 (0.5)	**<0.001**
Physical activity energy expenditure (kcal x kg^-1^ x d^-1^)	98.4 (32.4)	90.5 (28.1)	106 (34.3)	**<0.001**
Moderate to vigorous physical activity (min^-1^ x d^-1^)	115 (61.3)	97.5 (53.2)	132 (63.8)	**<0.001**
Finnish Children Healthy Eating Index	22.8 (7.0)	23.4 (6.5)	22.3 (7.2)	0.094

The data are mean (standard deviation) or median (interquartile range). P-values are for the difference between girls and boys from the Students t -test, Mann-Whitney U -test, or χ^2^ test. Statistically significant differences are bolded. LM, lean mass. Maturity was assessed as the difference between the current age from the age at predicted peak height velocity and was computed using a sex-specific formula described by Moore and coworkers (37).

### Associations of cardiorespiratory fitness with metabolic biomarkers

Cardiorespiratory fitness was directly associated with HDL cholesterol concentration, average HDL diameter, and the concentrations of extra-large, large, and medium size HDL particles as well as ApoA1, glutamine, and phenylalanine after adjustment for age and sex (**Table 2**). These associations remained after FDR_0.2_ correction, and even after FDR_0.1_ correction, except the association between cardiorespiratory fitness and the concentration of extra-large HDL particles. The direct associations of cardiorespiratory fitness with HDL cholesterol concentration, the concentration of medium size HDL particles, ApoA1, glutamine, and phenylalanine remained statistically significant even after further adjustment for BF%. Further adjustment for maturity, PAEE, MVPA, FCHEI, HOMA-IR, or protein intake had no effect on the magnitude of these associations (data not shown).

**Table 2:** Associations of cardiorespiratory fitness with NMR-derived metabolic biomarkers.

	Model 1		Model 2		BF% x CRF interaction
	β (95% CI)	p	β (95% CI)	p	p
**Metabolic biomarker**					
***Lipoproteins and triglycerides***					
VLDL cholesterol (mmol/l)	-0.022 (-0.118 to 0.075)	0.662	0.005 (-0.091 to 0.101)	0.922	0.136
LDL cholesterol (mmol/l)	-0.047 (-0.153 to 0.058)	0.379	0.027 (-0.070 to 0.124)	0.580	**0.017**
HDL cholesterol (mmol/l)	**0.138 (0.042 to 0.135)**	**0.005**	**0.114 (0.018 to 0.210)**	**0.021**	0.276
Total triglycerides (mmol/l)	0.024 (-0.073 to 0.122)	0.630	0.047 (-0.051 to 0.144)	0.349	0.868
Average VLDL diameter (nm)	0.001 (-0.096 to 0.090)	0.952	0.032 (-0.064 to 0.129)	0.510	0.856
Average LDL diameter (nm)	0.063 (-0.035 to 0.161)	0.204	0.062 (-0.037 to 0.161)	0.222	**0.030**
Average HDL diameter (nm)	**0.102 (0.004 to 0.199)**	**0.041**	0.051 (-0.034 to 0.155)	0.208	0.768
Concentration of medium VLDL particles (mmol/l)	-0.005 (-0.103 to 0.092)	0.913	0.024 (-0.073 to 0.120)	0.631	0.139
Concentration of small VLDL particles (mmol/l)	-0.009 (-0.107 to 0.088)	0.853	0.017 (-0.080 to 0.114)	0.731	0.508
Concentration of extra-small VLDL particles (mmol/l)	0.002 (-0.095 to 0.099)	0.968	0.012 (-0.085 to 0.110)	0.802	0.202
Concentration of extra-large HDL particles (mmol/l)	**0.103 (0.006 to 0.201)**	**0.038**	0.068 (-0.027 to 0.163)	0.161	0.940
Concentration of large HDL particles (mmol/l)	**0.122 (0.025 to 0.220)**	**0.014**	0.089 (-0.007 to 0.184)	0.069	0.730
Concentration of medium HDL particles (mmol/l)	**0.143 (0.047 to 0.239)**	**0.004**	**0.126 (0.030 to 0.223)**	**0.010**	0.224
Concentration of small HDL particles (mmol/l)	0.073 (-0.025 to 0.170)	0.143	0.095 (-0.002 to 0.192)	0.056	**0.020**
***Apolipoproteins***					
Apolipoprotein B (g/l)	0.003 (-0.094 to 0.101)	0.946	0.024 (-0.073 to 0.121)	0.630	0.029
Apolipoprotein A1 (g/l)	**0.145 (0.048 to 0.242)**	**0.003**	**0.126 (0.030 to 0.223)**	**0.011**	0.231
Apolipoprotein B to Apolipoprotein A1 ratio	-0.084 (-0.180 to 0.012)	0.086	-0.051 (-0.145 to 0.043)	0.287	0.180
***Amino acids***					
Alanine (mmol/l)	0.004 (-0.054 to 0.142)	0.377	0.061 (-0.038 to 0.159)	0.226	0.697
Glutamine (mmol/l)	**0.161 (0.064 to 0.257)**	**0.001**	**0.147 (0.050 to 0.244)**	**0.003**	0.387
Glycine (mmol/l)	0.068 (-0.030 to 0.165)	0.172	0.054 (-0.044 to 0.152)	0.278	0.919
Histidine (mmol/l)	0.079 (-0.118 to 0.177)	0.110	0.085 (-0.013 to 0.183)	0.090	0.578
Phenylalanine (mmol/l)	**0.187 (0.091 to 0.283)**	**<0.001**	**0.217 (0.122 to 0.311)**	**<0.001**	0.480
Tyrosine (mmol/l)	0.031 (-0.067 to 0.129)	0.537	0.060 (-0.036 to 0.157)	0.220	0.283
Isoleucine (mmol/l)	0.006 (-0.092 to 0.104)	0.904	0.019 (-0.079 to 0.118)	0.669	0.336
Leucine (mmol/l)	0.049 (-0.049 to 0.147)	0.325	0.071 (-0.027 to 0.169)	0.156	0.547
Valine (mmol/l)	0.042 (-0.056 to 0.140)	0.400	0.075 (-0.021 to 0.171)	0.126	0.630
Total branched-chain amino acids (mmol/l)	0.040 (-0.058 to 0.138)	0.426	0.067 (-0.030 to 0.164)	0.173	0.524

The data are standardised regression coeffiecients (β) and their 95% confidence intervals (95% CI) adjusted for age and sex (Model 1) and additionally for body fat percentage (model 2). P-values for statistically significant associations and interactions are bolded. BF%, body fat percentage; CRF, cardiorespiratory fitness; HDL, high density lipoprotein; LDL, low density lipoprotein; VLDL, very-low-density lipoprotein.

BF% modified the associations of cardiorespiratory fitness with LDL cholesterol concentration, average LDL diameter, and the concentrations of small HDL particles and ApoB (**Table 2**). Cardiorespiratory fitness was directly associated with average LDL diameter (β=0.198, 95% CI=0.062 to 0.334, p=0.004) and the concentration of small HDL particles (β=0.139, 95% CI=0.004 to 0.274, p=0.043) in children with higher BF%, but not in children with lower BF% (β=-0.074, 95% CI=-0.215 to 0.067, p=0.301, and β=-0.010, 95% CI=-0.130 to 0.151, p=0.887, respectively). Further adjustment for maturity, FCHEI, PAEE, MVPA, or HOMA-IR had no effect on the magnitude of these associations (data not shown). Other associations among children with higher or lower BF% were not statistically significant.

### Associations of cardiorespiratory fitness with HDL characteristics

The associations of cardiorespiratory fitness with HDL characteristics are presented in **Supplementary Table**. Cardiorespiratory fitness was directly associated with phospholipids, cholesteryl esters, free cholesterol, and total lipids in HDL. Cardiorespiratory fitness was also directly associated with total concentration, concentration of total lipids, phospholipids, cholesterol, cholesteryl esters, and free cholesterol of medium HDL particles and with the concentration of total lipids and phospholipids in small HDL particles after adjustment for age, sex, and BF%. All but one of these associations remained after FDR_0.2_ and FDR_0.1_ correction. The only exception was the association between cardiorespiratory fitness and phospholipids in extra-large HDL particles that did not remain after FDR_0.1_ correction. Further adjustments for maturity, PAEE, MVPA, and HOMA-IR had no effect on the magnitude of the associations. However, adjustment for FCHEI slightly attenuated the association of cardiorespiratory fitness with the concentration of total lipids (β=0.081, 95% CI=-0.016 to 0.179, p=0.103), phospholipids (β=0.092, 95% CI=-0.006 to 0.190, p=0.065), and free cholesterol (β=0.093, 95% CI=-0.005 to 0.192, p=0.063) in small HDL particles.

Sex modified the associations of cardiorespiratory fitness and some HDL particle characteristics (p ≤ 0.05 for cardiorespiratory fitness x sex interactions). Cardiorespiratory fitness was inversely associated with the proportion of phospholipids in large HDL particles (β=-0.149, 95% CI=-0.279 to -0.020, p=0.024) and proportion of cholesterol in large HDL particles (β=0.133, 95% CI=0.003 to 0.263, p=0.045) among boys, but not in girls (β=0.118, 95% CI=-0.021 to 0.257, p=0.095 and β=-0.125, 95% CI=-0.263 to 0.014, p=0.077, respectively). However, the inverse associations of cardiorespiratory fitness with HDL characteristics in boys were not statistically significant after further adjustment for BF% (β=0.100, 95% CI=-0.023 to 0.222, p=0.110 and β=0.095, 95% CI=-0.035 to 0.226, p=0.152, respectively).

BF% modified the associations between cardiorespiratory fitness with characteristics of small HDL particles. Cardiorespiratory fitness was directly associated with free cholesterol in small HDL particles in children with higher BF% (β=0.146, 95% CI=0.011 to 0.282, p=0.035), but not in children with lower BF% (β=0.034, 95% CI=0.107 to 0.175, p=0.633). Further adjustments had no effect on the magnitude of this association (data not shown).

## Discussion

We found that cardiorespiratory fitness was directly associated with serum HDL characteristics, ApoA1, glutamine, and phenylalanine in a general population of children. While adiposity explained part of the associations between cardiorespiratory fitness and HDL characteristics, most associations of cardiorespiratory fitness and metabolites were independent of adiposity, maturity, physical activity, diet quality, and insulin resistance. Cardiorespiratory fitness had weak if any associations with other lipoproteins, amino acids, or triglycerides. Furthermore, we found that adiposity modified the associations of cardiorespiratory fitness with average LDL diameter and the concentration of small HDL particles.

Higher cardiorespiratory fitness has been associated with higher HDL cholesterol, lower total cholesterol, lower total cholesterol to HDL cholesterol ratio, and lower triglycerides in children and adolescents (7), but the evidence remains mixed and thus prevents firm conclusions (8). Furthermore, higher V̇O_2max_ normalised for body mass has been associated with higher total cholesterol to HDL cholesterol ratio, while V̇O_2max_ normalised for lean body mass exhibited no association with blood lipids (9). This suggests that adiposity confounds the associations between cardiorespiratory fitness and blood lipids. However, traditional lipid measurements may not be sensitive enough to provide a full picture of the associations of cardiorespiratory fitness with atherogenic and cardioprotective blood lipid profile and previous studies have not used NMR metabolomics to assess blood HDL characteristics in children. We found consistent associations of cardiorespiratory fitness with HDL characteristics, but controlling for adiposity attenuated several of these associations. Nevertheless, several associations, particularly those with medium size HDL particle characteristics, were also independent of adiposity and other potential confounders. One reason for the positive associations between cardiorespiratory fitness and HDL characteristics could be the positive associations of HDL particles on cardiac and artery structures and functions. For example, HDL particles participate in endothelial vasodilatory functions by stimulating the release and production of nitric oxide from the artery wall (43), and arterial vasolidatory function is important for high cardiorespiratory fitness (44). Accordingly, a recent study in youth suggested that healthy arterial structures could enhance cardiorespiratory fitness (4). HDL may also directly or indirectly improve cardiac structures and functions and thereby increase cardiorespiratory fitness (27). Moreover, HDL has been found to improve cellular and mitochondrial functions (25, 26). Therefore, it is possible that HDL improves cardiovascular and skeletal muscle structures and functions that are central components of cardiorespiratory fitness. Finally, although the cross-sectional associations of cardiorespiratory fitness with HDL characteristics observed in our study were largely independent of current physical activity levels, these associations may reflect the effects of previous longer-term physical activity behaviour (45).

Children with higher adiposity exhibited a direct association between cardiorespiratory fitness and the concentration of small HDL particles. Duparc et al. (12) reported an inverse association between the concentration of small HDL particles and cardiovascular mortality. Small HDL particles have been suggested to provide cardioprotection through cholesterol efflux, antioxidant, anti-inflammatory, cytoprotective, and anti-thrombotic mechanisms (43, 46, 47). They also protect LDL from oxidation (47). Therefore, it is possible that higher concentration of small HDL particles along with higher cardiorespiratory fitness in children is an adaptive mechanism protecting against cardiovascular damage caused by increased adiposity. Consistently, we have previously found that physical activity is inversely associated with biomarkers of inflammation particularly in children with higher levels of adiposity (48). However, Shah et al. (17) found a direct association between the concentration of small HDL particles and pulse wave velocity in obese youth indicating a worse arterial health. It is possible that these latter observations reflect a decreased antioxidative capacity of small HDL particles observed in adults those with type 2 diabetes (49), suggesting that not only HDL particle size but also composition is important for arterial health. Nevertheless, it is yet to be investigated whether higher small HDL concentration improves cardiovascular health among children and adolescents with higher levels of adiposity and cardiorespiratory fitness compared to their peers with lower cardiorespiratory fitness.

In line with the studies in adults (24), we observed a direct association between cardiorespiratory fitness and ApoA1 concentration. ApoA1 is a main protein component of HDL and ApoA1 containing particles mediate the reverse cholesterol transport (50). Furthermore, a higher ApoA1 has been associated with better artery structure and function (51) suggesting that the mechanisms explaining these associations are probably similar than those of HDL characteristics (50). Furthermore, we did not find associations of cardiorespiratory fitness with LDL or VLDL characteristics. Previous studies in adults have observed such associations, but they have used measures of cardiorespiratory fitness that are confounded by adiposity, perhaps explaining the discrepancy between the results. Furthermore, we found that cardiorespiratory fitness was directly associated with average LDL diameter. Small dense LDL is a potent atherogenic lipoprotein (40, 52), and LDL particle size has been inversely associated with insulin resistance (17, 53). Therefore, it is possible that increased average LDL diameter would be seen as a cardioprotective response in children with higher BF% and increased insulin resistance (42). However, it remains unknown whether increased average LDL diameter would serve as a cardioprotective mechanisms between cardiorespiratory fitness and cardiovascular health.

We found that cardiorespiratory fitness was directly associated with amino acid glutamine in conjunction with some studies in adults (24). We also found, in contrast to the results by Kujala et al. (24), that the association was independent of BF%. One explanation why cardiorespiratory fitness is associated with glutamine may be that glutamine serves a precursor for l-arginine, that is used to synthetise a vasodilator nitric oxide (54, 55), which in turn is expected to improve cardiorespiratory fitness. It is also possible that an increased glutamine concentration reflects reduced systemic inflammation and oxidative stress (54, 55). The use of glutamine supplements has been associated with decreased markers of fatigue (56), that in turn may improve cardiorespiratory fitness. Taken together, these results suggest that cardiorespiratory fitness could be associated with cardioprotective and performance enhancing metabolic alterations related to amino acids metabolism. However, in contrast to adult studies, we found a positive association between cardiorespiratory fitness and phenylalanine (24) that has been directly associated with the risk of cardiovascular events (57). The reason for the direct association between cardiorespiratory fitness and phenylalanine in our study is unclear. However, the results of some studies suggest that l-phenylalanine could be associated with improved endothelial functions (58, 59), that could partly explain our finding. Nevertheless, more research is warranted to investigate the association between cardiorespiratory fitness and phenylalanine and its role in cardiovascular health since childhood.

While branched-chain amino acids have been associated with increased risk of cardiovascular disease in adults (15, 18), we did not find an association between cardiorespiratory fitness and these amino acids in children. This could be because branched-chain amino acids are more related to adiposity, as suggested by previous studies on the associations of adiposity and cardiorespiratory fitness with amino acids in adults (24). Therefore, our results together with previous studies suggest that the cardioprotective role of higher cardiorespiratory fitness is not explained by its effects on branched-chain amino acids.

The strengths of the present study include the valid and reproducible measurements of cardiorespiratory fitness using an exercise test until exhaustion, serum metabolic biomarkers using a NMR platform, and body composition using whole-body DXA in a population sample of children. We were also able to control for several confounding factors, including physical activity, diet quality, insulin resistance, and maturation. Nevertheless, we used W_max_ as a measure of cardiorespiratory fitness instead of directly measured V̇O_2max_. Additional studies investigating whether the associations of maximal oxygen uptake with serum metabolites are similar to those of peak performance with these metabolites are warranted. We also investigated only the associations between cardiorespiratory fitness and NMR-derived metabolic biomarkers and studies investigating whether HDL characteristics mediate the associations between cardiorespiratory fitness and arterial structures and functions. Finally, our study was cross-sectional which limits our ability to make causal inferences.

In conclusion, we found that higher cardiorespiratory fitness was associated with several cardioprotective NMR-derived metabolic biomarkers, especially those of related to HDL characteristics, independent of several confounding factors. Future studies are warranted to investigate whether the identified metabolites and especially those of HDL characteristics mediate the cardioprotective effects of cardiorespiratory fitness on arterial structures and functions since childhood.

## Data availability statement

The raw data supporting the conclusions of this article will be made available by the authors, without undue reservation.

## Ethics statement

The studies involving human participants were reviewed and approved by The Research Ethics Committee of the Hospital District of Northern Savo. Written informed consent to participate in this study was provided by the participants’ legal guardian/next of kin.

## Author contributions

EH, MHL, ML, US, TL participated the conception of the study. TT, AV, NL, US, TL collected the data. EH conducted the analyses and produced the first draft of the manuscript. All authors participated in drafting and revising the manuscript and provided significant intellectual contribution to the manuscript, and all authors approved the final version of the manuscript. All authors agree to be accountable for the work and to ensure that any questions relating to the accuracy and integrity of the paper are investigated and properly resolved. All authors contributed to the article and approved the submitted version.

## Funding

The PANIC Study has financially been supported by Ministry of Education and Culture of Finland, Ministry of Social Affairs and Health of Finland, Research Committee of the Kuopio University Hospital Catchment Area (State Research Funding), Finnish Innovation Fund Sitra, Social Insurance Institution of Finland, Finnish Cultural Foundation, Foundation for Paediatric Research, Diabetes Research Foundation in Finland, Finnish Foundation for Cardiovascular Research, Juho Vainio Foundation, Paavo Nurmi Foundation, Yrjö Jahnsson Foundation, and the city of Kuopio. Moreover, the PhD students and postdoctoral researchers of The PANIC Study have been supported by Program for Clinical Research and Program for Health Sciences of Doctoral School of University of Eastern Finland, Finnish Doctoral Programs in Public Health, Päivikki and Sakari Sohlberg Foundation, Paulo Foundation, Jalmari and Rauha Ahokas Foundation, Aarne and Aili Turunen Foundation, Finnish Medical Foundation, Jenny and Antti Wihuri Foundation, Kuopio Naturalists’ Society, Olvi Foundation, and the city of Kuopio. EH has been funded by the Juho Vaino foundation and the Finnish Foundation for Cardiovascular Research. The sponsors had no role in designing the study, the collection, analysis, or interpretation of the data, the writing of the report, or the decision to submit the manuscript for publication.

## Acknowledgments

We are grateful to the members of the PANIC research team for their contribution in acquisition of data. We are also indebted to all children and their parents participating in the PANIC Study.

## Conflict of interest

The authors declare that the research was conducted in the absence of any commercial or financial relationships that could be construed as a potential conflict of interest.

## Publisher’s note

All claims expressed in this article are solely those of the authors and do not necessarily represent those of their affiliated organizations, or those of the publisher, the editors and the reviewers. Any product that may be evaluated in this article, or claim that may be made by its manufacturer, is not guaranteed or endorsed by the publisher.
