# Supplementary material for: Cross-sectional associations between cardiorespiratory fitness and NMR-derived metabolic biomarkers in children – the PANIC study
Source: Front Endocrinol (Lausanne). 2022 Sep 23;13:954418. doi: 10.3389/fendo.2022.954418 (PMC9538338; doi:10.3389/fendo.2022.954418)
Supplement: Supplementary file 1 [file Table_1.docx]

| **Supplementary Table**. Associations of cardiorespiratory fitness with the HDL characteristics in children. | | | | | |
| --- | --- | --- | --- | --- | --- |
|  | **Model 1** |  | **Model 2** |  | **BF% x CRF**  **interaction** |
|  | **β (95% CI)** | **p** | **β (95% CI)** | **p** | **p** |
| **Metabolic biomarker** |  |  |  |  |  |
| ***Characteristics of HDL particles*** |  |  |  |  |  |
| Triglycerides in HDL particles (mmol/l) | 0.061 (-0.037 to 0.159) | 0.221 | 0.048 (-0.051 to 0.148) | 0.341 | 0.837 |
| Phospholipids in HDL particles (mmol/l) | **0.144 (0.047 to 0.240)** | **0.004** | **0.123 (0.026 to 0.219)** | **0.013** | 0.310 |
| Cholesteryl esters in HDL particles | **0.138 (0.041 to 0.234)** | **0.005** | **0.113 (0.017 to 0.210)** | **0.021** | 0.265 |
| Free cholesterol in HDL particles (mmol/l) | **0.138 (0.041 to 0.235)** | **0.006** | **0.113 (0.017 to 0.210)** | **0.022** | 0.331 |
| Total lipids in HDL particles (mmol/l) | **0.143 (0.047 to 0.240)** | **0.004** | **0.121 (0.025 to 0.217)** | **0.014** | 0.290 |
| Total lipids in extra-large HDL particles (mmol/l) | 0.097 (-0.004 to 0.195) | 0.051 | 0.061 (-0.035 to 0.156) | 0.213 | 0.890 |
| Phospholipids in extra-large HDL particles (mmol/l) | **0.100 (0.002 to 0.198)** | **0.045** | 0.062 (-0.033 to 0.157) | 0.199 | 0.826 |
| Cholesterol in extra-large HDL particles (mmol/l) | 0.092 (-0.006 to 0.190) | 0.066 | 0.056 (-0.040 to 0.152) | 0.250 | 0.969 |
| Cholesteryl esters in extra-large HDL particles (mmol/l) | 0.097 (-0.003 to 0.195) | 0.051 | 0.062 (-0.034 to 0.157) | 0.205 | 0.942 |
| Free cholesterol in extra-large HDL particles (mmol/l) | 0.067 (-0.031 to 0.165) | 0.182 | 0.031 (-0.064 to 0.127) | 0.519 | 0.649 |
| Triglycerides in extra-large HDL particles (mmol/l) | 0.096 (-0.002 to 0.194) | 0.054 | 0.077 (-0.020 to 0.175) | 0.120 | 0.938 |
| Total lipids in large HDL particles (mmol/l) | **0.134 (0.027 to 0.221)** | **0.013** | 0.090 (-0.005 to 0.185) | 0.064 | 0.726 |
| Phospholipids in large HDL particles (mmol/l) | **0.126 (0.029 to 0.223)** | **0.011** | 0.093 (-0.003 to 0.188) | 0.057 | 0.730 |
| Cholesterol in large HDL particles (mmol/l) | **0.120 (0.023 to 0.217)** | **0.016** | 0.086 (-0.010 to 0.181) | 0.078 | 0.720 |
| Cholesteryl esters in large HDL particles (mmol/l) | **0.120 (0.023 to 0.218)** | **0.015** | 0.087 (-0.009 to 0.182) | 0.075 | 0.706 |
| Free cholesterol in large HDL particles (mmol/l) | **0.117 (0.020 to 0.225)** | **0.019** | 0.083 (-0.013 to 0.178) | 0.089 | 0.770 |
| Triglycerides in large HDL particles (mmol/l) | **0.109 (0.012 to 0.207)** | **0.028** | 0.089 (-0.008 to 0.187) | 0.073 | 0.884 |
| Total lipids in medium HDL particles (mmol/l) | **0.142 (0.046 to 0.239)** | **0.004** | **0.129 (0.032 to 0.226)** | **0.009** | 0.200 |
| Phospholipids in medium HDL particles (mmol/l) | **0.138 (0.042 to 0.235)** | **0.005** | **0.128 (0.031 to 0.224)** | **0.010** | 0.213 |
| Cholesterol in medium HDL particles (mmol/l) | **0.143 (0.046 to 0.239)** | **0.004** | **0.125 (0.125 to 0.222)** | **0.011** | 0.191 |
| Cholesteryl esters in medium HDL particles (mmol/l) | **0.142 (0.046 to 0.238)** | **0.004** | **0.125 (0.028 to 0.221)** | **0.011** | 0.193 |
| Free cholesterol in medium HDL particles (mmol/l) | **0.142 (0.046 to 0.239)** | **0.004** | **0.127 (0.030 to 0.0224)** | **0.011** | 0.193 |
| Triglycerides in medium HDL particles (mmol/l) | 0.051 (-0.047 to 0.149) | 0.306 | 0.068 (-0.037 to 0.160) | 0.219 | 0.816 |
| Total lipids in small HDL particles (mmol/l) | 0.076 (-0.022 to 0.173) | 0.128 | **0.098 (0.001 to 0.195)** | **0.048** | **0.039** |
| Phospholipids in small HDL particles (mmol/l) | 0.093 (-0.004 to 0.190) | 0.061 | **0.110 (0.012 to 0.207)** | **0.027** | 0.059 |
| Cholesterol in small HDL particles (mmol/l) | 0.054 (-0.043 to 0.152) | 0.276 | 0.081 (-0.016 to 0.178) | 0.101 | **0.018** |
| Cholesteryl esters in small HDL particles (mmol/l) | 0.040 (-0.058 to 0.137) | 0.423 | 0.068 (-0.029 to 0.165) | 0.167 | **0.020** |
| Free cholesterol in small HDL particles (mmol/l) | 0.091 (-0.007 to 0.189) | 0.067 | 0.109 (0.011 to 0.207) | **0.029** | **0.033** |
| Triglycerides in small HDL particles (mmol/l) | -0.010 (-0.108 to 0.088) | 0.835 | 0.015 (-0.082 to 0.113) | 0.756 | 0.877 |
|  |  |  | Continues next page | | |
| **Supplementary Table continues.** | **Model 1** |  | **Model 2** |  | **BF% x CRF**  **interaction** |
|  | **β (95% CI)** | **p** | **β (95% CI)** | **p** | **p** |
| **Metabolic biomarker** |  |  |  |  |  |
| ***Characteristics of HDL particles*** |  |  |  |  |  |
| Phospholipids/total lipids ratio in extra-large HDL particles (%) | 0.065 (-0.033 to 0.163) | 0.192 | 0.016 (-0.077 to 0.109) | 0.735 | 0.343 |
| Cholesterol/total lipids ratio in extra-large HDL particles (%) | -0.080 (-0.177 to 0.018) | 0.110 | -0.033 (-0.126 to 0.060) | 0.485 | 0.487 |
| Cholesteryl esters/total lipids ratio in extra-large HDL particles (%) | -0.045 (-0.143 to 0.054) | 0.373 | -0.003 (-0.098 to 0.092) | 0.949 | 0.202 |
| Free cholesterol/total lipids ratio in extra-large HDL particles (%) | **-0.104 (-0.202 to -0.007)** | **0.035** | -0.065 (-0.159 to 0.293) | 0.176 | 0.840 |
| Triglycerides/total lipids ratio in extra-large HDL particles (%) | 0.016 (-0.081 to 0.114) | 0.743 | 0.044 (-0.053 to 0.141) | 0.370 | 0.325 |
| Phospholipids/total lipids ratio in large HDL (%) | -0.017 (-0.114 to 0.081) | 0.736 | 0.010 (-0.087 to 0.107) | 0.840 | 0.723 |
| Cholesterol/total lipids ratio in large HDL particles (%) | 0.003 (-0.095 to 0.101) | 0.954 | -0.021 (-0.119 to 0.076) | 0.669 | 0.851 |
| Cholesteryl esters/total lipids ratio in large HDL particles (%) | 0.004 (-0.095 to 0.102) | 0.943 | -0.016 (0.114 to 0.082) | 0.750 | 0.794 |
| Free cholesterol/total lipids ratio in large HDL particles (%) | -0.002 (-0.098 to 0.097) | 0.0985 | -0.034 (-0.130 to 0.062) | 0.486 | 0.857 |
| Triglycerides/total lipids ratio in large HDL particles (%) | 0.023 (-0.075 to 0.121) | 0.639 | 0.038 (-0.060 to 0.136) | 0.448 | 0.882 |
| Phospholipids/total lipids ratio in medium HDL particles (%) | -0.046 (-0.145 to 0.052) | 0.353 | -0.023 (-0.121 to 0.075) | 0.644 | 0.606 |
| Cholesterol/total lipids ratio in medium HDL particles (%) | 0.029 (-0.070 to 0.127) | 0.568 | 0.007 (-0.091 to 0.104) | 0.896 | 0.614 |
| Cholesteryl esters/total lipids ratio in medium HDL particles (%) | 0.007 (-0.091 to 0.105) | 0.886 | -0.011 (-0.109 to 0.088) | 0.830 | 0.769 |
| Free cholesterol/total lipids ratio in medium HDL particles (%) | **0.109 (0.011 to 0.207)** | **0.029** | 0.084 (-0.013 to 0.181) | 0.089 | 0.260 |
| Triglycerides/total lipids ratio in medium HDL particles (%) | -0.009 (-0.107 to 0.089) | 0.859 | 0.010 (-0.088 to 0.108) | 0.839 | 0.648 |
| Phospholipids/total lipids ratio in small HDL particles (%) | **0.120 (0.023 to 0.217)** | **0.015** | 0.094 (-0.002 to 0.190) | 0.055 | 0.666 |
| Cholesterol/total lipids ratio in small HDL particles (%) | -0.088 (-0.185 to 0.001) | 0.078 | -0.076 (-0.174 to 0.223) | 0.129 | 0.412 |
| Cholesteryl esters/total lipids ratio in small HDL particles (%) | -0.093 (-0.191 to 0.005) | 0.062 | -0.078 (-0.176 to 0.020) | 0.118 | 0.430 |
| Free cholesterol/total lipids ratio in small HDL particles (%) | 0.052 (-0.045 to 0.148) | 0.297 | 0.034 (-0.063 to 0.131) | 0.487 | 0.879 |
| Triglycerides/total lipids ratio in small HDL particles (%) | -0.035 (-0.133 to 0.063) | 0.483 | -0.014 (-0.111 to 0.840) | 0.785 | 0.390 |

The data are standardised regression coeffiecients (β) and their 95% confidence intervals (95% CI) adjusted for age and sex (Model 1) and additionally for body fat percentage (model 2). P-values for statistically significant associations and interactions are bolded. BF%: body fat percentage; CRF: cardiorespiratory fitness; HDL: high density lipoprotein.
